# Preprints in Medicine: Useful or Harmful?

**DOI:** 10.3389/fmed.2020.579100

**Published:** 2020-09-22

**Authors:** Bruno Bonnechère

**Affiliations:** ^1^Department of Psychiatry and Behavioral and Clinical Neurosciences, University of Cambridge, Cambridge, United Kingdom; ^2^Public Health School, Université Libre de Bruxelles, Brussels, Belgium

**Keywords:** preprint, publications, data repository, public, publication science

## Introduction

Research and its associated publications have had a considerable impact on the care and monitoring of the patients since evidence-based medicine became standard for modern medicine during the 1990s (1). Peer-reviewing is a fundamental component of scientific publication. The peer-review process first includes an evaluation of the quality and interest in the paper for the reader of the journal by the editor who, if he or she considers the article to be of interest, sends it to the external reviewers (2). If the paper is found to be interesting and of sufficient quality, the reviewers ask questions and make comments to which the researcher must respond in a rebuttal letter. If the answers are satisfactory, the article can be published. This is a time-consuming process, typically lasting months, and authors complain about the review time, which has been relatively stable since the 1980s (3).

## The Evolution of Scientific Publications

In previous decades, the world of scientific publishing has changed enormously with an explosion in the number of publishers and journals. Therefore, there has been an exponential increase in the number of scientific papers published (4). The way that information is distributed has also changed, with an increasing number of journals being available in open access format. In this model, the cost of the publication is paid by the authors, and the manuscript is then freely available once accepted for publication. Traditionally there is no cost to publish the paper; the publishers sell articles or subscription systems through university libraries.

There are pros and cons to open access format, which are not going to be discussed here as they are outside the scope of this paper. However, from an individual's point of view, this is an interesting evolution, enabling readers to keep up to date with scientific research without having to take out a subscription.

In recent years, another phenomenon that has been developing is also having an increasing impact on research: preprints. The preprint is a version of a scientific paper that precedes the peer review process, and the article is freely available without any form of control by the editor or reviewers. Furthermore, most preprints receive a digital object identifier (DOI). Authors can, therefore, reference them in their bibliography or grant applications. Subsequent revisions are time-stamped, and anyone can read and comment on the paper. This collaborative aspect is often put forward by the proponents of this model.

Initially developed in the field of physics and economy (e.g., arXiv, RePEc), this practice is now spreading to the medical world and other domains of research. Preprints were the fastest-growing of all types of research output (around 30%) in 2016–2018, compared with article growth of 2–3% (5).

Although preprint publishers have a legal obligation to indicate, in the article, that it is a draft that has not been peer-reviewed, the recent COVID-19 pandemic has highlighted the ambiguity raised by this practice in medicine.

In this paper, we discuss the impact of preprints on the general population, the media, and research more generally from the researchers' and publishers' points of view. We also present different solutions to improve the current publication model that ensure the authorship of innovation while guaranteeing the quality and accuracy of publications.

## Public Impact

Before going further, it must be said that the COVID-19 pandemic is an exceptional situation that was unprecedented for the general population at large. This crisis has highlighted the role of dissemination of research to the general public but has also demonstrated some limitations of the current research model or, at least, the dissemination of the results.

With maximum media coverage, and in the absence of effective treatment, journalists were on the lookout for the slightest piece of information, verified or not. The information contained in preprints has been spread to the general public by both official and unofficial media, without reference to their distinction from peer-reviewed papers. Preprints and media are not the only ones to blame as numerous publication scandals have erupted during this crisis which, in the context of fake news and misinformation, has certainly not helped the recognition of research (6) and the confidence in research process by the public (7).

In this context, the public has been confused by the mass of information available. We can cite, as examples, some of the most striking cases of this crisis: a genomic study of the virus carried out by an Indian team who had discovered similarities with HIV, opening the door to a conspiracy theory, which was then withdrawn by the authors after significant methodological errors were identified by the readers of the preprint (8). The most famous examples are the publication scandal related to the danger of hydroxychloroquine that were detailed in the *Lancet* (9) and the *New England Journal of Medicine* (10).

It should be pointed out, however, that two of the three examples given above-involved articles published in peer-reviewed and high-impact journals—proof that the review process is not perfect and does not always guarantee the accuracy of the results (11). During the COVID-19 crisis, due to political pressure for fast publication, most of the journals implemented a fast-track review process (i.e., a shorter period of time to review the paper). It is challenging for the reviewers to check the plausibility and quality of the data and statistics, which could explain the scandals and the retractions.

## The Researcher Point of View

Modern research can be summarized by two words: novelty and innovation (12). Scientists promote preprints because they enable researchers to claim priority (i.e., intellectual property) and make their findings available more quickly (13). Another advantage of preprints was demonstrated by a recent study showing that articles with a preprint have, on average, a 49% higher Altmetric Attention Score and 36% more citations than articles without a preprint (14). Since most of preprints have a DOI, some researchers use preprints to artificially boost their bibliography for grant applications or promotions.

Innovation is, of course, the raison d'être of scientific research. That being said, by promoting innovation above all, there is a great risk that the authors will want to rush their studies and draw incomplete or incorrect conclusions (15). This phenomenon was accentuated during the crisis, during which intermediate and incomplete results of clinical studies were published as a result of political pressure. The authors also wanted to make themselves known in order to submit special projects related to the COVID.

On the other hand, an essential aspect of the research is the protection of novelty using patents. Inventions can be patented if they are novel, non-obvious, and useful (16). When an invention is publicly disclosed, it enters into state of the art. Consequently, no one will be able to patent the same invention as the novelty requirement has been impeded (17). Prior art, the material publicly available prior to the filing date of the patent, includes all the different sources of publications, it therefore also includes the preprints. This can have a substantial negative effect on the translation of an invention into an innovation that has the potential to reach the patient. Indeed, if an invention cannot be patented, it loses the interest of the private companies. Even if researchers are less comfortable with writing patents, they should therefore first patent innovation before publishing anything related to this, whatever the publication is peer-reviewed, preprint, or event conference presentation, to foster innovation (18).

Paradoxically, while the number of published papers is growing exponentially, it seems that it has become more and more challenging to reach a consensus within the scientific community, and the results of published studies are difficult to reproduce. Some authors argue, therefore, that there is currently a reproducibility crisis as most scientists have failed to reproduce the results of published papers (19, 20). With the rapid publication of incomplete and unverified results, preprints are contributing to a significant increase in the quantity of research, often difficult to replicate due to vague or incomplete description of the protocol and the low quality of the results.

These findings are particularly compelling in the field of healthcare, where treatment decisions are made based on the results of clinical studies (21), especially since results of preprints are now included in some meta-analyses (22). Replicated studies should not be undervalued by editors and should receive the attention they deserved to solve this reproducibility crisis (23).

Another useful way to protect novelty and innovation is to make data available for other researchers (24). A growing number of journals require authors to share data by depositing them in a repository when submitting their manuscripts. This is beneficial not only in ensuring the correct analysis and interpretation of the results but also to ensure that the data are used to their fullest potential for improving individual and public health (25). Some ethical questions of sharing issues can, however, arise—especially—when private companies are involved in the research (e.g., commercial use of personal data) (26). Therefore, this practice needs to be well-supervised, and the data must be fully protected.

## From a Publisher Perspective

For the publishers, three aspects of preprints need to be considered: the content, the repository (i.e., publication and distribution), and the financial impact.

Concerning the content, some journals, such as the *New England Journal of Medicine* or *Science*, view draft preprints as prior publications (prior art, see above) and, thus, they are unacceptable as new manuscript submissions (5).

Some publishers (e.g., MDPI, JMIR Publications) have their own preprint repository, allowing for better synchronization between the preprint and final versions of the paper. Ideally, preprints should be removed once the article has been accepted or rejected.

Concerning the financial impact, we noticed that preprints often stay online, which represents a potential loss of income for the publishers and can be confusing if the final version of the published paper contains substantial changes compared with the first (preprint) version. On the other hand, we have seen that papers with a preprint have more citations that papers without preprint (14), which represents an increase in visibility, and therefore potentially in the revenues generated.

## Conclusion and Call For Action

As a scientist, the increasingly widespread use of preprints is an excellent opportunity to question the current limits of the peer review process. Publication scandals are not uncommon, even in high impact factor journals (although this can be biased because those journals are more widely read and analyzed, so we cannot confirm that scandals are more common specifically for top journals) (27). Solutions to increase the quality of peer-reviewing have been proposed: single vs. double-blind peer review (28) or open peer review (29).

Between financial interests, on the one hand, and personal stakes (i.e., the need to publish) on the other, the publishing world is a key factor in research. It would, therefore, be utopian to promote the end of preprints, especially since we have seen that it increases the visibility and citations of papers, which is good for both researchers and publishers. Rather than abolishing preprints, we are instead advocating a stricter framework for this practice, and better information to the public and the media on the differences between preprints and peer-reviewed papers.

We suggest that preprints are published only once the manuscript is under review in one journal, indicating that it has at least been screened and checked by an editor, and that it is indicated in the preprint in which journal the paper has been submitted.

By doing so, the authors retain the advantage of the preprint—speed of publication and previous tracing in cases of conflict (i.e., intellectual property but authors should be aware that preprints are part of the prior art)—as well as saving time and money by avoiding duplication, with at least a first control by the journal and the editor.

After the review process, the preprint should be removed in the case of acceptance in order to leave only the final version online. Scientific literature is already so prolific that it is necessary to avoid having to take duplicates between preprints and published papers into account. In the case of rejection, the preprint should also be withdrawn, since the reviewers felt that the paper could not be published for various reasons (e.g., methodological issues).

Finally, we do not think that preprints should receive a DOI, or at least, that it should not be used for grant submission. It is certain that this aspect of preprints leads to the most abuse and ultimately undermines the quality and reputation of research.

By applying these few recommendations, the positive aspects of preprints are preserved for the authors, and the quality of the published papers will be preserved without creating ambiguity in the general public.

## Author Contributions

BB wrote the manuscript.

## Conflict of Interest

The author declares that the research was conducted in the absence of any commercial or financial relationships that could be construed as a potential conflict of interest.
